# Design of a process evaluation of the implementation of a physical activity and sports stimulation programme in Dutch rehabilitation setting: ReSpAct

**DOI:** 10.1186/s13012-014-0127-7

**Published:** 2014-09-20

**Authors:** Femke Hoekstra, Roelina A Alingh, Cees P van der Schans, Florentina J Hettinga, Marjo Duijf, Rienk Dekker, Lucas HV van der Woude

**Affiliations:** University of Groningen, University Medical Center Groningen, Center for Human Movement Sciences, Groningen, The Netherlands; University Medical Center Groningen, Center for Rehabilitation, Department of Rehabilitation Medicine, Groningen, The Netherlands; University Medical Center Groningen, Center for Sports Medicine, Groningen, The Netherlands; Hanze University of Applied Sciences, Research and Innovation Group in Health Care and Nursing, Groningen, The Netherlands; University of Essex, School of Biological Sciences, Centre of Sport and Exercise Science, Colchester, UK; Stichting Onbeperkt Sportief, Bunnik, The Netherlands

**Keywords:** Implementation, Dissemination, Rehabilitation, Physical activity, Active lifestyle, Process evaluation, Disability, Chronic disease, Health promotion

## Abstract

**Background:**

There is a growing interest to study the transfer of evidence-based information into daily practice. The evidence-based programme Rehabilitation, Sports and Exercise (RSE) that aims to stimulate an active lifestyle during and after a rehabilitation period in people with a disability and/or chronic disease is prepared for nationwide dissemination. So far, however, little is known about the implementation of a new programme to stimulate physical activity in people with a disability in a rehabilitation setting. Therefore, a process evaluation of the implementation of the RSE programme within 18 Dutch rehabilitation centres and hospitals is performed in order to gain more insight into the implementation process itself and factors that facilitate or hamper the implementation process. This paper describes the study design of this process evaluation.

**Methods:**

During a three-year period, the adoption, implementation and continuation of the RSE programme is monitored and evaluated in 12 rehabilitation centres and 6 hospitals with a rehabilitation department in The Netherlands. The main process outcomes are: recruitment, reach, dose delivered, dose received, fidelity, satisfaction, maintenance and context. The process outcomes are evaluated at different levels (organisational and patient) and different time points. Data collection includes both quantitative (online registration system and questionnaires) and qualitative (focus groups and semi-structured interviews) methods.

**Discussion:**

The nationwide dissemination of an evidence-based programme to stimulate physical activity and sports during and after a rehabilitation period is extensively monitored and evaluated on different levels (organization and patients) using mixed methods. The study will contribute to the science of translating evidence-based programmes into daily practice of the rehabilitation care. The results of the study can be used to further optimize the content of the RSE programme and to facilitate the implementation in other health facilities. Furthermore, the results of the study can help future implementation processes in the rehabilitation setting.

**Trial registration:**

The study is registered by The Netherlands National Trial Register: NTR3961.

**Electronic supplementary material:**

The online version of this article (doi:10.1186/s13012-014-0127-7) contains supplementary material, which is available to authorized users.

## Background

Throughout the last decades, much attention has been given to the development of programmes that aim to stimulate an active lifestyle in people with or without a disability and/or chronic disease [1]-[4]. The literature showed promising results with regard to the improvements on physical activity behaviour in different population groups [1],[2],[5]. In most cases, such programmes or interventions have been studied under controlled conditions, rather than in the real world [3],[6]. It appears, however, that the step to a real-world setting is complex and often fails. Therefore, researchers have shown a growing interest in the need to study the transfer of interventions into daily practice and to understand the factors that are associated with a successful or unsuccessful transfer.

There are several steps in the transfer of an evidence-based intervention into daily practice [7]-[9]. Throughout this process, the organizations, including the involved professionals, have to go through three main steps. During the first main step (adoption), the professionals in the organization decide that they want to work with the new intervention. During the second step (implementation), the intervention is implemented into the organization and delivered to the persons concerned. In the last step (continuation), the intervention is integrated into the organization and maintained over time. During each step, the process is influenced by different factors, both positively and negatively [10],[11].

Identifying factors that facilitate or hamper the adoption, implementation and continuation of a new programme is important for a successful implementation process. It has been shown that a successful implementation of a new programme is associated with better results of the programme on the individual level [12]. Therefore, an evaluation of the implementation process of a new programme can help facilitate understanding and explanation of the results of the programme [13].

Several researchers identified factors that lead to successful application of a new programme. For example, Wierenga *et al.*[14] published a review on factors that facilitate or hamper the implementation of a health promotion programme at the workplace. Furthermore, others have performed a Delphi study and identified factors that are relevant for the adoption, implementation and continuation of a physical activity intervention in the primary healthcare [11]. The authors also highlighted the importance of paying attention to the different steps of the implementation process and identified factors that are specifically relevant for these steps [11]. So far, however, little is known about the implementation of a programme to stimulate physical activity in people with a disability. Moreover, even less scientific research is performed on the identification of factors that influence this process in the context of a rehabilitation treatment.

It has been proposed that the ideal timing of promoting an active lifestyle in people with a disability is immediately after the rehabilitation treatment [4],[15]. The authors suggested that promoting participation in physical activities and sports immediately after the rehabilitation period would also provide the opportunity to close the existing gap between the rehabilitation setting and the sports and exercise facilities in the community [15]. The suggestion to stimulate an active lifestyle after rehabilitation was studied in a randomized controlled trial by van der Ploeg *et al.*[16]. These authors investigated the effects of two programmes to promote physical activity and sports participation in people with a disability. The results of the study showed that patients who participated in the combined sports and active lifestyle stimulation programme developed a better daily physical activity and sports behaviour compared to patients who participated in the sports stimulation programme and the control group. The effects were visible on both the short [16] and long term [17] (9 and 52 weeks after the end of an inpatient or outpatient rehabilitation). Therefore, during the following years (2009 to 2011), this evidence-based intervention was further developed and prepared for nationwide dissemination by the Dutch Foundation `Stichting Onbeperkt Sportief’^a^. As a result, the new intervention, which is called `Revalidatie, Sport en Bewegen’ (in English: `Rehabilitation, Sports and Exercise’ [RSE]), is currently being implemented in rehabilitation centres and hospitals with a rehabilitation department in The Netherlands.

The study is part of the nationwide ReSpAct (Rehabilitation, Sports and Active Lifestyle) study [18]. This paper presents the study design of the process evaluation of the adoption, implementation and continuation of the RSE programme within 18 Dutch rehabilitation centres and hospitals. Therefore, the aim of the current study is to describe the design of the process evaluation of the implementation of the RSE programme within 18 Dutch rehabilitation centres and hospitals in order to gain more insight into the implementation process itself and factors that facilitate or hamper the implementation process.

## Methods

### Study design

The ReSpAct study is a multicenter longitudinal cohort study, in which data are collected in a real-world setting on different levels (organization and patient). During a three-year period, the implementation of the RSE programme is monitored and evaluated in 12 rehabilitation centres and 6 hospitals with a rehabilitation department in The Netherlands. For a successful implementation process, it is not only important that the programme is effective at the level of the patient, but also that the implementation strategy fits with the context of the organization [19],[20]. Therefore, the current process evaluation focuses on components related to the content of the RSE programme and on the implementation strategy throughout the whole implementation period. Data collection includes both quantitative (online registration system and questionnaires) and qualitative (focus groups and semi-structured interviews) methods in a repeated measures set-up. The use of a combination of quantitative and qualitative data creates a rich dataset and a complete overview of the process outcomes, which makes it possible to gain better understanding in the implementation process and the related determinants.

### Organizations and study population

The RSE programme is being implemented in 12 rehabilitation centres and 6 hospitals with a rehabilitation department in The Netherlands from October 2012 to December 2015. All 18 organizations are receiving financial and advisory assistance to support the implementation process. Furthermore, all participating organizations are included in the process evaluation. The programme developer (`Stichting Onbeperkt Sportief’) was responsible for the recruitment of the centres and hospitals. If a rehabilitation centre or hospital was interested in the implementation of the RSE programme, the programme coordinator of the RSE programme visited the organization and explained the implementation procedures. Furthermore, the current situation and the ambitions with respect to the integration of exercise and sports into the rehabilitation treatment were inventoried. An important goal was to include centres and hospitals located across the different regions of The Netherlands in order to control for possible regional variations. If the organization met the criteria to participate, they were invited to sign a declaration to participate in the programme in order to formalize the adoption of the programme. The inclusion criteria for organizations were as follows:

1. Sufficient support for the RSE programme from the professionals of the organization;
2. Sufficient ambition to integrate exercise and sports into the rehabilitation treatment;
3. Sufficient intention to continue the RSE programme after the project period.

After signing this declaration, the programme coordinator discussed the procedures of the programme and requirements for participation in the ReSpact study in more detail with the professionals in the concerning organization. During this stage, the centres and hospitals were instructed to make an organization-specific project plan for the implementation and continuation of the RSE programme. The participation of the organization in the RSE programme was formalized by signing an agreement to participate by the head of the organization. By signing this document, the organization made, theoretically, the step from the adoption to the implementation of the RSE programme. This document included the following elements:

1. willingness to implement the RSE programme according to the protocol during a period of three years (2012 - 2015);
2. willingness to participate in the ReSpAct study until December 2015;
3. willingness to maintain the RSE programme after December 2015.

Rehabilitation centres that participated as intervention centres in the previous study of van der Ploeg *et al.*[17] were excluded from participation. The main reason for this exclusion criterion was to give other (and sometimes smaller) rehabilitation centres and hospitals in the Netherlands the opportunity to implement the RSE programme by being given financial and advisory support.

To collect data on the level of the organization, all managers, project leaders, physicians and counsellors who are involved in the implementation of the RSE programme in their centre or hospital are asked to participate in the process evaluation. To collect extensive data on the individual level, patients who participate in the RSE programme are also asked to enrol into the ReSpAct study. It is aimed to recruit 2,000 adult patients in total from all 18 organizations together. Counsellors in the involved centres and hospitals are responsible for the recruitment of patients for the ReSpAct study. The inclusion criteria for patients are: a physical disability and/or chronic disease, a minimum age of 18 years, and receiving treatment at one of the participating rehabilitation centres or hospitals. This treatment can consist of an inpatient or outpatient rehabilitation or a treatment based on medicine consultation. Inability to fill out the questionnaires that are part of the ReSpAct study was the only exclusion criterion.

### The programme `rehabilitation, sports, and exercise’

The RSE programme was developed by the Dutch organization `Stichting Onbeperkt Sportief’ and is a tailored counselling programme based on the results of the evidence-based combined physical activity and sports stimulation programme of the study of van der Ploeg *et al.*[17]. The programme aims to stimulate a physically active lifestyle in people with a physical disability and/or a chronic disease during and after their rehabilitation period. In order to establish a behavioural change, all consultations that are part of the RSE programme are based on motivational interviewing (MI) [21]. The `Physical Activity for people with a Disability model’ (PAD model) [22] was used as a theoretical framework providing the basis for the understanding of the outcomes of the programme at the level of the patient. A detailed description of the evaluation of the RSE programme at the patient level is described elsewhere [unpublished study protocol by Alingh R.A., Hoekstra, F., Schans, C.P. van der, Hettinga, F.J., Dekker, R., Woude, L.H.V. van der].

The RSE programme consists of the following main components:

1. Intake session on exercise and sports

An intake session is used to identify wishes and interests with regard to exercise and sports participation of the patient and is a standard component of the rehabilitation treatment. In this way, individual goals with respect to the exercise and sports activities during rehabilitation can be formulated within the individual treatment plan. The intake session can take place with a physician or another therapist who is involved in the rehabilitation treatment programme.

2. Exercise and sports are standard components of the rehabilitation treatment

The centres and hospitals integrate exercise and sports activities as a standard component of an individual rehabilitation treatment programme. The organisation of exercise and sports clinics for people with a physical disability and/or chronic disease can be part of this component. In this way, patients can be introduced into various exercise and sports activities as part of their rehabilitation period.

3. Referral to Sports Counselling Centre

Part of the RSE programme is setting up a Sports Counselling Centre (SCC) within the rehabilitation centre or hospital. The SCC is a specific department in the organization where the consultations of RSE programme take place. Three to six weeks before the end of the rehabilitation, patients are referred to the SCC of the rehabilitation centre or hospital. The physical activity and sports counsellors working at the SCC are health professionals specialized in (adapted) physical activity and/or physiotherapy and trained in MI. The counsellor gives patients support and advice in finding and engaging in physical activities, exercise and/or sports activities in the home setting. Within each rehabilitation centre and hospital, the procedure of referring patients to the SCC should be clear and well organized.

4. Face-to-face consultation

After the referral to the SCC, patients receive an individual face-to-face consultation with a counsellor to support and stimulate an active lifestyle at home. The counsellor gives tailored advice with regard to the participation in daily physical activities, exercise and/or sports activities in the home setting. A referral to an exercise or sports activity in the region can also be part of this advice. The sessions are based on MI [21].

5. Four telephone-based counselling sessions

After the end of rehabilitation treatment, patients receive four counselling sessions by phone with the counsellor of the SCC. During these counselling sessions, patients are further supported and stimulated in realizing and maintaining a physically active lifestyle at home.

6. Collaboration between SCC and external exercise and sports facilities (network)

In order to provide tailored advice for exercise and sports participation, counsellors need to know which exercise and sports activities in the region are accessible for people with a physical disability and/or chronic disease. Building up a network between the rehabilitation centre/hospital and external exercise/sports facilities is therefore an important component of the RSE programme. As a result, the SCC will establish a link between the rehabilitation health care and the regional network of exercise/sport activities in the Netherlands.

Furthermore, if the exercise and sports facilities in the region are not sufficient for people with a disability and/or chronic disease, the rehabilitation centre or hospital itself is suggested to organize exercise and sports activities for this population. These activities can be seen as a supplement to the exercise and sports facilities in the community.

### Implementation strategy for the RSE programme

The implementation strategy that is used for the dissemination of the RSE programme consists of different components to support the implementation and continuation of the RSE programme in the participating organizations. This practical implementation strategy includes components that can contribute to a successful implementation process [10], such as collaboration and training.

The main component of the implementation strategy consists of regular visits of the two programme coordinators of `Stichting Onbeperkt Sportief’ to the participating centres and hospitals in order to coordinate and support the adoption, implementation and continuation. During these visits, the programme coordinators intend to meet all members of the project group. Therefore, the organizations are advised to form a project group directly after they decided to adopt the RSE programme. Furthermore, professionals in the participating organizations write project plans, annual plans and annual reports concerning the implementation and execution of the RSE programme. The programme coordinators review these plans and reports and provide feedback. In addition, throughout the whole project period, the programme coordinators and the ReSpAct research team are also available to answer questions and/or give advice on the implementation and executing of the RSE programme in the participating organizations. Furthermore, the websites of the RSE programme [23] and ReSpAct study [24] provide general information about the RSE programme and the ReSpAct study, the newsletters, participating organizations, and relevant contact information.

In order to facilitate the communication between organizations, a minimum of two national or regional meetings are organized by the programme developers and the ReSpAct research team each year. During these meetings, professionals in the participating organizations have the opportunity to share knowledge and their experiences. Some group discussions during these days are also used to gain more insight into the implementation process within the different organizations and the possible determinants of implementation. In addition to the meetings and visits, an internet forum is available for professionals to share knowledge and experiences as well as to ask questions.

As part of the implementation strategy, a three-day training course for MI is offered to all counsellors in the participating rehabilitation centres and hospitals. During this course, the basic principles and skills of MI are explained and trained. In addition to the standard course, an annual return-day was organized for the counsellors to refresh and deepen their MI skills.

To further support the implementation process, the rehabilitation centres and hospitals received a `Handbook’ for the implementation of the RSE programme [25]. This book includes detailed information and instructions about the main components of the RSE programme. Furthermore, the book gives an overview of different steps that the organizations have to take to implement the RSE programme in their own organization. The steps are based on practical experiences and described as follows:

1. Analysis of the starting position of the rehabilitation centre/hospital

In the first place, centres and hospitals should determine their own starting positions related to the different components of the RSE programme (*e.g*., the extent to which exercise and sports is part of the rehabilitation treatment).

2. Develop and set goals for the organization

After determining the starting position, centres and hospitals should set goals related to the main components of the RSE programme (intake session, exercise and sports during rehabilitation, referral to SCC, face-to-face consultations, counselling sessions, collaboration between SCC and external exercise and sports facilities) [25].

3. Analysis of possibilities to collaborate

Collaboration with other professionals within and between organizations (*e.g*., other rehabilitations centres/hospitals and external exercise and sports facilities) can facilitate the implementation process. An analysis of possible partners to collaborate is therefore recommended.

4. Develop an action plan including time planning

During the fourth step, all activities that have to be performed should be described in a detailed action plan. From this document, an annual plan should be derived.

5. Monitor and evaluation

Finally, the centre/hospital should monitor and evaluate its own process. Part of this step is to write an annual report.

### The theoretical framework of the process evaluation

The implementation process of the RSE programme will be evaluated by using the recently published theoretical framework [14]. In this framework, different theoretical models [7],[9],[10],[13] are combined into one theoretical framework. As a result, the evaluation will be done in a systematic way and will include process outcomes that are not only related to the dose and reach of the programme, but also to the fidelity and satisfaction of the programme. Moreover, special attention will be given to the context in which the implementation takes place. According to the framework, the following central aspects can be identified:

1. Implementation process: adoption
2. Implementation process: implementation
3. Implementation process: continuation
4. Implementation determinants

**Figure 1:**
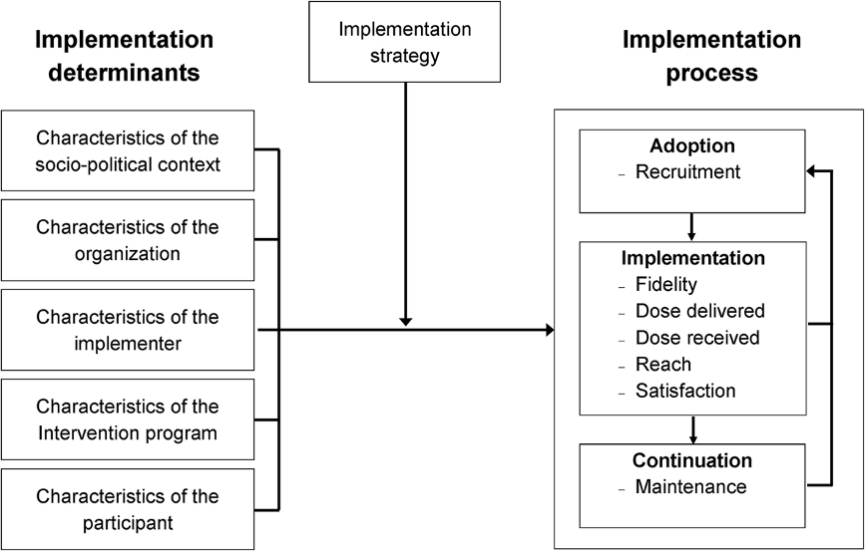
**Theoretical framework adapted from Wierenga** ***et al.*** **[**[14]**].**

These four aspects need to be assessed by using eight process outcomes (recruitment, fidelity, dose delivered, dose received, reach, satisfaction, maintenance and context) that will be evaluated on different organizational levels [14],[26] (see Table 1 and Figure 1). In the current study, data collection started after the rehabilitation centres and hospitals decided to adopt the RSE programme. Therefore, the aspects related to the adoption will only be assessed retrospectively. As a consequence, the focus of the current process evaluation will be on the implementation and continuation of the RSE programme and on the related determinants. Table 1 presents the outcomes that are measured during the implementation process of the RSE programme on both the level of the organization and the patient.

**Table 1:** **Process outcomes of the evaluation of the implementation process of the RSE programme**

Process outcomes	Definitions of process outcomes*	Description	Data collection
1) Adoption			
*Recruitment:*	`Procedures used to approach centres and hospitals to participate in the RSE programme’.	Organization level:	I
		- Strategy of inviting organizations to participate in the RSE programme	
		- Reasons of organizations for (not) participating in the programme	
2) Implementation			
*Fidelity:*	*`* The extent to which the RSE programme has been implemented as planned (the quality of the implementation)’.	Organization level:	Q, RS, I, FG
		- Conformity to the implementation strategy (main components)	
		- Conformity to the RSE programme (main components)	
*Dose delivered*	`The amount of the RSE programme that is delivered or performed by the professionals’.	Organization level:	Q, RS
		- Amount of activities performed as part of the implementation strategy	
		- Amount of introductory sessions delivered	
		- Amount of sport and exercise activities as standard components of the rehabilitation treatment	
		- Amount of face-to-face sessions delivered by the counsellor	
		- Amount of counselling sessions delivered by the counsellor	
		- Amount of collaborations with exercise and sport facilitators (network)	
*Dose received*	`The amount of the RSE programme that is received by the patients’.	Patient level:	Q, RS
		- Number/percentage of patients who get acquainted with sport and exercise activities during rehabilitation treatment	
		- Number/percentage of patients who are referred to the SCC	
		- Number/percentage of patients who received a face-to-face consultation	
		- Number/percentage of patients who received counselling	
		- Number/percentage of patients who are referred to a sport and exercise activity in the region	
*Reach*	`The extent to which professionals and persons with a physical disability and/or chronic disease are reached by the implementation of the RSE programme’.	Organization level:	Q, RS, FG
		- Number of exercise and sport facilities that collaborate with participating organizations (network)	
		- Number/percentage of professionals participating in activities that are part of the implementation strategy	
		- Number/percentage of professionals participating in the RSE programme	
		Patient level:	
		- Number/percentage of patients participating in the RSE programme	
*Satisfaction*	`Opinion about the RSE programme and the implementation strategy’.	Organization level:	Q, RS, FG
		- Opinion about the implementation strategy by professionals	
		- Opinion about the content of the RSE programme by professionals	
		- Satisfaction about the implementation RSE programme within the organization	
		Patient level:	
		- Satisfaction/opinion about the RSE programme by patients	
		- Satisfaction about the sport and exercise facilities in the region by patients	
3) Continuation			
*Maintenance*	`The extent to which the RSE programme is integrated into the routines and into the organization’.	Organization level:	Q, I, FG
		- The integration of the RSE programme into the standard rehabilitation treatment	
		- The integration of the RSE programme into the policy of the organization	
4) Implementation determinants			
*Context*	`Aspects of the environment that influence the implementation of the RSE programme or the RSE programme outcomes’.	Organization and patient level:	Q, RS, I, FG
		- Characteristics of the social-political context	
		- Characteristics of the rehabilitation centre/hospital	
		- Characteristics of the professionals of the centre/hospitals	
		- Characteristics of the RSE programme	
		- Characteristics of the patients	

*Definitions are based on the literature of Steckler and Linnan [13] and Saunders *et al.*[33]; Q = questionnaires, RS = registration system, I = interviews, FG = focus groups.

As can be seen in Figure 1, the potential factors that facilitate or hamper the implementation process (`implementation determinants’) can be classified into the following groups:

1. Characteristics of the social-political context
2. Characteristics of the rehabilitation centre/hospital
3. Characteristics of the professionals of the centres/ hospital
4. Characteristics of the RSE programme
5. Characteristics of the patients

The other block of the framework is the implementation strategy, which is also an essential element to successfully implement a new programme into daily practice [19]. The components related to the implementation strategy of the RSE programme as described in the previous section are also incorporated in the process outcomes (see Table 1 and Figure 1).

### Data collection

**Figure 2:**
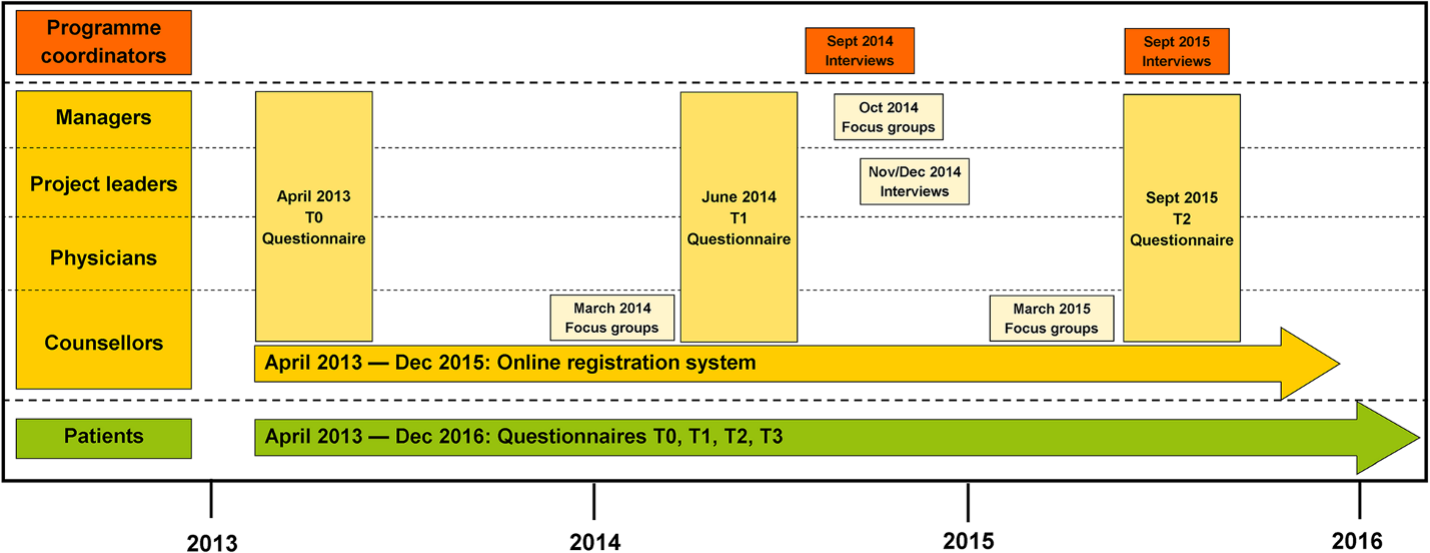
**Overview of the different methods used to collect data for the process evaluation.** Data are collected at different levels and at different time points.

The evaluation of the implementation process of the RSE programme will be performed over a period of three years. Quantitative and qualitative data are collected on the level of the organization and the patient level. A complete overview of the different methods that are being used to collect the data that at the different levels is shown in Figure 2.

#### Level of the organization: questionnaires

Managers, project leaders, physicians and counsellors are asked to fill out a questionnaire at baseline (April 2013), one year later (June 2014), and at the end of the implementation period (September 2015). The expected numbers of professionals participating in the process evaluation are: 18 managers, 18 project leaders, 18 physicians and approximately 36 counsellors. The theory-based questionnaires were constructed based on the literature of Fleuren *et al*. [10] and Grol *et al.*[8]. The questionnaires include questions about the different process outcomes (*i.e*., fidelity, dose, reach, satisfaction, and context) and possible factors that hamper or facilitate the implementation process (Table 1). The questionnaires not only contain questions with multiple choice answers (4- or 5-point Likert scale) but also open-ended questions.

In order to provide questions related to the tasks of the different professionals, different questionnaires were constructed and adapted to the different professions. This procedure resulted in four different questionnaires for four different groups of professionals: managers, project leaders, physicians and counsellors. When a professional fulfils more than one role (*i.e*., manager and project leader), both questionnaires are combined. Filling out the questionnaire takes approximately 30 to 45 minutes, depending on the role(s) of the professional.

#### Level of the organization: online registration system

An online registration system was developed to collect real-time data about the dose, reach and fidelity of the RSE programme. The counsellors are asked to complete an online form after each face-to-face consultation delivered at the SCC. This form includes questions about basic characteristics of the concerning patient (year of birth, gender, disease/disability, type of rehabilitation treatment) and some questions about the content of the face-to-face conversations. The aim of this registration form is to collect real-time data about the total number of patients who participated in the RSE programme including some basic characteristics of the patients and delivered consultations. Completing this form lasted approximately two minutes.

In addition to this, counsellors complete a more extensive form about dose delivered to patients who gave written informed consent to participate in the ReSpAct study. This form includes questions about the date, duration, mode and content of all consultations between counsellor and patient (face-to-face and counselling sessions). Based on this information, more insight can be gained about the extent to which the RSE programme has been delivered as planned. Completing this extensive form lasted approximately 15 minutes.

#### Level of the organization: focus group discussions and semi-structured interviews

All managers who are involved in the implementation of the RSE programme are invited to participate in a focus group discussion about financial aspects related to the implementation and execution of the RSE programme. The available financial resources are an important determinant for a successful implementation and continuation of the RSE programme. Possibilities to receive financial support from health insurances, local authorities and/or other organizations are discussed with the involved managers. Qualitative techniques will be used to detect possible factors that hamper or facilitate the implementation and continuation of the RSE programme.

Similarly, a focus group discussion is organized for the involved counsellors in the participating centres and hospitals. During this session the content of the RSE programme is discussed with the counsellors. Special attention is given to their opinion about the RSE programme and possibilities to improve the content of the RSE programme.

Furthermore, all project leaders (n = 18) are asked for a semi-structured interview to collect data about the fidelity and satisfaction of the implementation strategy and content of the RSE programme. Also, the experienced factors that hamper and facilitate the implementation process are discussed during the interviews. The qualitative data gathered from the interviews will be used to detect, explain and interpret factors leading to success and failure.

Finally, the programme coordinators (n = 2) are asked for a semi-structured interview during and at the end of the implementation period to gain more insight into the delivered support to the individual centres and hospitals. Also, their experiences and satisfaction about the adoption, implementation and continuation of the RSE programme are discussed. Qualitative data obtained from the interviews with the programme coordinators will be used to verify and/or explain the findings of the process evaluation.

#### Patient level: questionnaires

Patients participating in the ReSpAct study (n = 2,000) fill out a questionnaire at baseline, 14 weeks after the end of the rehabilitation period and at a 33 and 52 weeks follow-up. The questionnaires include questions about quality of life, physical activity behaviour and its related determinants (*i.e*., attitude, self-efficacy, social support, barriers and facilitators). The health-related quality of life will be assessed by using the adapted version of the RAND-36 [27]-[29]. The SQUASH questionnaire, adjusted for patients with a physical disability, is used to measure the physical activity behaviour of the patients [30]. Furthermore, patients are asked about their opinion and experiences of the received support from the counsellor of the SCC. Detailed descriptions of the content of the questionnaires with respect to the physical activity outcomes are described elsewhere [unpublished study protocol by Alingh *et al*.]. Dose-response relationships are used to gain more insight into the effectiveness of the RSE programme and its underlying mechanisms.

### Data analyses

The evaluation of the implementation process will be performed by using the eight process outcomes as described in Table 1. The process outcomes will include both quantitative and qualitative data. Descriptive analyses (frequencies, means and percentages) of the quantitative data collected from the online registrations system and questionnaires will be performed with SPSS version 20.0 (SPSS Inc. Chicago, Illinois, USA). The qualitative data collected during the interviews and focus groups will be audiotaped and transcribed verbatim. After reading the transcripts several times to familiarize with the text, codes will be identified. Subsequently, these codes will be categorized into different themes [31]. Qualitative data analyses will be performed by using the software programme `Atlas.ti.’

### Ethical considerations

The Medical Ethical Committee of the University Medical Center Groningen has exempted the approval of the study protocol. Therefore, the ethics committee of the Center of Human Movement Sciences of the University Medical Center Groningen approved the study protocol at the levels of both the organization and the patients.

### Trial status

The implementation process in the 18 participating rehabilitation centres and hospitals is being monitored until December 2015. Data collection on the level of the organization started in April 2013 and is ongoing until December 2015. The recruitment of the patients to participate in the ReSpAct study is ongoing and will stop at the end of 2015, indicating that data collection on patient level is ongoing until the end of 2016.

## Discussion

In the last decades, much research has been performed on translating evidence-based programmes into daily practice [3],[32]. Performing a process evaluation can be helpful to gain more insight into factors that hamper or facilitate this translation to daily practice [13],[33]. This paper described the design of the process evaluation of a unique nationwide dissemination of an evidence-based programme to stimulate physical activity and sports during and after a rehabilitation period (the RSE programme). This study design is the first step of sharing our ambitions, knowledge and plans with regard to the integration and stimulation of physical activity, exercise and sports in the Dutch rehabilitation setting.

The present study will therefore contribute to the science of translating evidence-based programmes into daily practice of the rehabilitation care. By using the theoretical framework of Wierenga *et al.*[14] to evaluate the implementation of the RSE programme, information on the implementation process will be obtained and evaluated on different levels and during the entire implementation period. This will result in a rich dataset that will expand the knowledge on the translation of new programmes into daily practice. Furthermore, the theoretical framework is also presented in an evaluation of the implementation of a lifestyle intervention at the workplace [26]. Therefore, the present study allows a better insight into the application possibilities of this framework in a health care setting.

Furthermore, the implementation process of the RSE programme is unique, because 12 Dutch rehabilitation centres and 6 hospitals with a rehabilitation department are involved and are situated across the country. In 2010, The Netherlands comprised 21 rehabilitation centres and 81 hospitals that offered a rehabilitation treatment [34]. Because a relatively large number of the total Dutch rehabilitation care organisations is involved in the present project, it is expected that the dissemination and evaluation of the RSE programme will have a large nationwide impact on the Dutch rehabilitation care. Furthermore, during the implementation of the RSE programme, participating organizations will build up a network with external sports and exercise facilities for people with a disability and/or chronic disease. As a result, it is expected that the dissemination of the RSE programme will establish the link between the rehabilitation care and exercise/sport facilities in the community throughout The Netherlands [15].

A main strength of the current study design is that the implementation process of the RSE programme is extensively monitored and evaluated on different levels (patients and organizations) and by using mixed methods. Furthermore, data is being collected over a three-year period, which makes it possible to evaluate the process outcomes longitudinally. The use of a combination of quantitative and qualitative methods will allow us to verify and combine the results by using triangulation [35],[36]. The use of mixed methods at different time points will therefore contribute to a complete and better understanding of the results on both the level of the organization and the patient.

Another strength of this study is that simultaneous to the process evaluation, a study to evaluate the programme outcomes is performed [18]. Although this design creates more work for the involved professionals, there are advantages of carrying out these two evaluations simultaneously. Early research has shown that a successful implementation of a new programme is associated with better results of the programme on the individual level [12]. When measuring and evaluating the RSE programme outcomes and process outcomes simultaneously, it is possible to investigate how they relate to each other. In this way, the results of the process evaluation can help us to understand and explain the outcomes of the programme on the level of the patient [13],[33].

Another strong point of the implementation process itself is that the process is coordinated and supported by the programme developers. The participating organizations are thus receiving financial, material and advisory support during the implementation period. Moreover, the practical implementation strategy includes activities that have been shown to contribute to successful implementation of a new programme into daily practice [10],[11]. Furthermore, the implementation of the RSE is supported by the Netherlands Society of Physical and Rehabilitation Medicine. This society has established an accredited working group on exercise and sports that aims to integrate exercise and sports into the rehabilitation in order to support an active lifestyle in persons with a disability during and after the rehabilitation period, which is in line with the aims of the RSE programme. Consequently, the dissemination of the RSE programme in 18 Dutch rehabilitation centres and hospitals has large potential to be successful.

There are also some limitations that should be mentioned. The inclusion criteria that are formulated for the organizations included that there was sufficient support and ambition by the professionals in the organization to implement the RSE programme. Although these criteria are important factors for a successful implementation process [10], it might have biased the sample of the participating organizations. It is possible that the participating rehabilitation centres and hospitals are more willing to implement the RSE programme compared to the other Dutch rehabilitation centres and hospitals. This possible recruitment bias should be taken into account when analysing the results of the process evaluation.

Another limitation of the present study is that the process evaluation is performed in the Dutch rehabilitation setting. The organization structure of the rehabilitation care in The Netherlands is relatively well organized and can differ from other countries. For example, before the start of the implementation of the RSE programme, exercise and sports were already to some extent integrated into the Dutch rehabilitation care [17]. Therefore, it is important to realize that the results of the present study cannot directly be applied to rehabilitation care outside The Netherlands. Despite the fact that direct application of the results may not be possible in all countries, the organization of the Dutch rehabilitation care can be used as an example to organizations in other countries. When analysing and discussing the results of the study, it is valuable to pay attention to the specific context in which the data are collected and to discuss the application possibilities of the results in a different context.

### Practical relevance

The present study will be relevant for daily practice. This study will identify factors that hamper or facilitate the implementation of a new programme in rehabilitation centres and hospitals. It has been shown that these factors may vary in different contexts [10]. The information achieved in this study can be used in future projects in which new programmes or interventions are implemented in a rehabilitation setting.

Moreover, the data collected during this process evaluation can be used to further optimize the content of the RSE programme. Based on the information that is collected from both the professionals and the patients, specific recommendations can be formulated to optimize the content of the RSE programme. It is likely that such optimizations will improve the programme outcomes at patient level.

### Endnotes

^a^Stichting Onbeperkt Sportief is an organization that aims for a larger participation within disabled sports and physical activity and the development of suitable and accessible sports facilities.

## Authors’ contributions

All authors contributed to the design and protocol of the study. LHVW, CPS, RD, FJH and MD contributed to obtaining funding. FH drafted the manuscript. LHVW, RAA, CPS, FJH, MD and RD reviewed the manuscript and provided comments and revisions. All authors read and approved the final manuscript.

## Authors’ original submitted files for images

Below are the links to the authors’ original submitted files for images. Authors’ original file for figure 1 Authors’ original file for figure 2

## Abbreviations

MI: Motivational Interviewing
RSE: Rehabilitation, Sports and Exercise
ReSpAct: Rehabilitation, Sports and Active Lifestyle
SCC: Sports Counselling Centre
