# Supplementary figures and images for: Design of a process evaluation of the implementation of a physical activity and sports stimulation programme in Dutch rehabilitation setting: ReSpAct
Source: Implement Sci. 2014 Sep 20;9:127. doi: 10.1186/s13012-014-0127-7 (PMC4177248; doi:10.1186/s13012-014-0127-7)

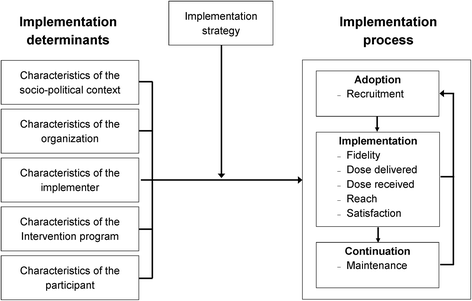

Supplement: Supplementary file 1 — Authors’ original file for figure 1 [file 13012_2014_127_MOESM1_ESM.gif]

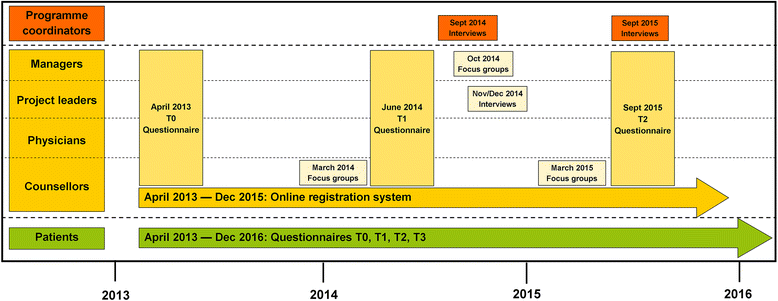

Supplement: Supplementary file 2 — Authors’ original file for figure 2 [file 13012_2014_127_MOESM2_ESM.gif]
